# High *RAD54B* expression: an independent predictor of postoperative distant recurrence in colorectal cancer patients

**DOI:** 10.18632/oncotarget.4222

**Published:** 2015-05-20

**Authors:** Yuzo Nagai, Yoko Yamamoto, Takaaki Yasuhara, Keisuke Hata, Takeshi Nishikawa, Toshiaki Tanaka, Junichiro Tanaka, Tomomichi Kiyomatsu, Kazushige Kawai, Hiroaki Nozawa, Shinsuke Kazama, Hironori Yamaguchi, Soichiro Ishihara, Eiji Sunami, Takeharu Yamanaka, Kiyoshi Miyagawa, Toshiaki Watanabe

**Affiliations:** ^1^ Department of Surgical Oncology, Faculty of Medicine, The University of Tokyo, Hongo, Bunkyo-ku, Tokyo, Japan; ^2^ Laboratory of Molecular Radiology, Center for Disease Biology and Integrative Medicine, Graduate School of Medicine, The University of Tokyo, Hongo, Bunkyo-ku, Tokyo, Japan; ^3^ Department of Biostatistics, Graduate School of Medicine, Yokohama City University, Suehiro-cho, Tsurumi-ku, Yokohama, Kanagawa, Japan

**Keywords:** RAD54B, colorectal cancer, homologous recombination, prognosis, distant recurrence

## Abstract

We recently reported a specific mechanism that RAD54B, an important factor in homologous recombination, promotes genomic instability via the degradation of p53 protein *in vitro*. However, clinical significance of RAD54Bin colorectal cancer (CRC) remains unclear. Thus we analyzed *RAD54B* geneexpression in CRC patients. Using the training set (n = 123), the optimal cut-off value for stratification was determined, and validated in another cohort (n = 89). Kaplan–Meier plots showed that distant recurrence free survival was significantly lesser in high *RAD54B* expression group compared with that of low expression group in both training (*P* = 0.0013) and validation (*P* = 0.024) set. Multivariate analysis using Cox proportional-hazards model showed that high *RAD54B* expression was an independent predictor in both training (hazard ratio, 4.31; 95% CI, 1.53–13.1; *P* = 0.0060) and validation (hazard ratio, 3.63; 95% CI, 1.23–10.7; *P* = 0.021) set. In addition, a negative significant correlation between *RAD54B* and *CDKN1A*, *a* target gene of p53, was partially confirmed, suggesting that RAD54B functions via the degradation of p53 protein even in clinical samples. This study first demonstrated RAD54B expression has potential to serve as a novel prognostic biomarker, particularly for distant recurrence in CRC patients.

## INTRODUCTION

Despite the recent progress in the therapeutic modalities, colorectal cancer (CRC) is a serious public health issue worldwide owing to its high incidence and cancer-related mortality [1]. In addition to TNM staging system, several biomarkers are currently applied to practical use for CRC patients. For example, UGT1A1 genetic polymorphism is associated with CPT-11 toxicity [2]. KRAS/NRAS mutations status predicts response to anti-EGFR antibody therapy [3, 4]. CRC with microsatellite instability has a better prognosis compared to CRC with intact mismatch repair [5]. Such biomarkers have improved clinical outcomes in CRC, however, the number of biomarkers still remains insufficient, and more studies exploring new biomarkers are required to establish further personalized therapeutic strategy.

Human *RAD54B* was first identified in 1999 as a homolog of *RAD54*. RAD54B belongs to SNF2/SWI2 superfamily, and biochemical studies have shown that RAD54B is involved in the homologous recombination (HR) [6-9]. HR is one of the most important DNA double-strand break repair pathway. It is generally accepted that imbalanced regulation of the HR system is associated with genomic instability, resulting in carcinogenesis and malignant tumor development [10-12].

Recently, our group found another mechanism different from the HR repair pathway in multiple cell line experiments, including the HCT116 colon cancer cell line. RAD54B is associated with cell cycle control and functions as a scaffold for p53 degradation through its direct interaction with the MDM2–MDMX ubiquitin–ligase complex, and constitutive upregulation of RAD54B activity promotes genomic instability [13]. Although one study concerning the effectiveness of capecitabine and concurrent radiation therapy for glioblastoma analyzed the expression profiles of eight genes including *RAD54B* and reported that high *RAD54B* expression was associated with a poor outcome [14], the clinical significance of *RAD54B* expression remains unknown particularly in CRC.

Therefore, we examined *RAD54B* expression in surgically resected CRC tissues by real-time PCR method and analyzed the correlation with various clinicopathological factors and patient's prognosis. In addition to *RAD54B*, we also analyzed *RAD51* expression in the same cohort because RAD51 is another important factor involved in HR process [15-18] and several studies reported that RAD51 protein overexpression is associated with poor prognosis in various cancers [19-24]. Moreover, we measured *CDKN1A (p21/WAF1)* expression, a target gene of p53, and analyzed the association between *RAD54B* and *CDKN1A* expression to investigate the biological role of *RAD54B* in clinical samples. From a clinical perspective, this is the first study demonstrating the utility of *RAD54B* as a prognostic biomarker in CRC patients.

## RESULTS

### *RAD54B* expression was elevated in most CRC tissues

We first analyzed *RAD54B* and *RAD51* expression in the training set (*n* = 123). Gene expression of *RAD54B*, *RAD51 were quantified by* real-time PCR as described in the Materials and Methods section. Table 1 shows the summary of *RAD54B* and *RAD51* expression values in the training set. The median (inter-quartile range) *RAD54B* expression value was 2.60 (2.50–3.99), and *RAD54B* expression values were higher than 1.00 in 116 of the 123 samples (94.3%), indicating that *RAD54B* expression was elevated in most CRC tissues compared with corresponding normal mucosa. In contrast, *RAD51* expression value [1.15 (0.80–1.56)] was not as elevated as *RAD54B*. The number of samples with values higher than 1.00 was 74 of the 123 samples (60.2%), which was significantly smaller than that of *RAD54B* (*P* = 0.0008).

**Table 1:** *RAD54B* and *RAD51* expression values in the training set (n = 123)

Category	No.	*RAD54B* value	No.	*RAD51* value	*P* value
All patients		2.60 (2.50-3.99)	1.15 (0.80-1.56)		
Expression value <1.00	7 (5.69%)		49 (39.8%)		0.0008
Expression value ≥1.00	116 (94.3%)		74 (60.2%)		

### *RAD54B* and *RAD51* expression values and clinicopathological factors in the training set

We next analyzed the relationship between *RAD54B* or *RAD51* expression and clinicopathological features in the training set. As shown in Table 2, no statistically significant correlations were found between *RAD54B* expression values and clinicopathological factors, such as sex, age, tumor location, tumor size, cell differentiation, lymphatic invasion, venous invasion, and preoperative CEA and CA19-9 levels. Similarly, no significant correlations were detected between *RAD54B* expression values and UICC stage (*P* = 0.20), T stage (*P* = 0.16), and N stage (*P* = 0.23). Likewise, there was no association between *RAD51* expression values and clinicopathological factors including UICC stage (*P* = 0.078), T stage (*P* = 0.51), and N stage (*P* = 0.38), except that *RAD51* expression values in lymphatic invasion-positive patients were significantly higher than those in negative patients (*P* = 0.027).

**Table 2:** Clinicopathological factors and *RAD54B* and *RAD51* expression values in the training set (n = 123)

Category		No (%)	*RAD54B* value	P value	*RAD51* value	P value
Sex	Male	65 (52.8)	2.46 (1.52-3.60)	0.11	1.02 (0.71-1.50)	0.65
	Female	58 (47.2)	2.78 (2.16-4.16)		1.23 (0.84-1.62)	
Age	<65	56 (45.5)	2.72 (1.97-4.19)	0.91	1.14 (0.81-1.56)	0.97
	≥65	67 (54.5)	2.50 (2.10-3.65)		1.15 (0.77-1.57)	
Tumor location	Colon	96 (78.0)	2.69 (2.05-4.22)	0.28	1.19 (0.79-1.58)	0.34
	Rectum	27 (22.0)	2.42 (1.97-3.40)		1.02 (0.80-1.48)	
Tumor size (mm)	<50	66 (53.7)	2.48 (2.05-3.24)	0.12	1.17 (0.80-1.53)	0.93
	≥50	57 (46.3)	2.88 (1.98-1.73)		1.05 (0.78-1.58)	
Cell differentiation	WD	57 (46.3)	2.48 (1.98-3.33)	0.42	1.20 (0.79-1.56)	0.68
	MD	62 (50.4)	2.62 (2.08-4.33)		1.11 (0.78-1.57)	
	PD	4 (3.3)	4.47 (1.97-5.40)		1.50 (0.82-2.74)	
Lymphatic invasion	Positive	47 (38.2)	2.66 (1.57-4.57)	0.92	1.35 (0.97-1.79)	0.027
	Negative	76 (61.8)	2.56 (2.14-3.69)		1.01 (0.79-1.48)	
Venous invasion	Positive	27 (22.0)	2.58 (2.00-4.12)	0.94	1.13 (0.80-1.57)	0.97
	Negative	96 (78.0)	2.56 (2.05-3.41)		1.18 (0.79-1.53)	
Preoperative CEA	<5.0	61 (49.6)	2.44 (1.87-4.11)	0.67	1.17 (0.80-1.56	0.28
	≥5.0	61 (49.6)	2.72 (2.10-3.82)		1.14 (0.79-1.56)	
Preoperative CA19-9	<37	99 (80.5)	2.56 (1.98-3.78)	0.59	1.10 (0.80-1.56)	0.26
	≥37	23 (18.7)	2.97 (2.10-4.54)		1.35 (0.69-1.93)	
Tumor stage (UICC)	I	13 (10.6)	2.58 (2.13-3.04)	0.20	1.09 (0.76-1.38)	0.078
	II	51(41.5)	2.72 (223-4.45)		1.18 (0.80-1.56)	
	III	44 (35.8)	2.42 (1.43-3.59)		1.02 (0.73-1.53)	
	IV	15 (12.2)	2.98 (2.11-5.26)		1.56 (1.13-2.67)	
T stage	T1	5 (4.1)	2.21 (1.05-5.31)	0.16	0.68 (0.36-9.42)	0.51
	T2	18 (14.6)	2.76 (2.08-3.03)		1.09 (0.87-1.40)	
	T3	60 (48.8)	2.44 (1.62-3.94)		1.13 (0.78-1.53)	
	T4	40 (32.5)	2.87 (2.18-5.32)		1.28 (0.81-1.79)	
N stage	N0	67 (54.5)	2.72 (2.21-3.87)	0.23	1.15 (0.80-1.51)	0.38
	N1	47 (38.2)	2.46 (1.53-3.42)		1.04 (0.78-2.28)	
	N2	9 (7.3)	4.54 (1.77-4.00)		1.39 (1.11-1.76)	
Distant Recurrence in Stage I-III	Yes	15 (12.2)	3.87 (2.55-4.88)	0.031	1.16 (0.81-1.79)	0.69
	No	93 (75.6)	2.46 (1.92-3.24)		1.07 (0.78-1.52)	

WD = well differentiated; MD = moderately differentiated; PD = poorly differentiated.

### *RAD54B* and *RAD51* expression and patient prognosis in the training set

Among the 108 stage I–III CRC patients (excluding stage IV patients) in the training set, 15 patients developed distant recurrence during the median follow-up period of 50.7 (40.9–59.9) months (Table 2). *RAD54B* expression values in patients developing distant recurrence were significantly higher than those in patients without distant recurrence [3.87 (2.55–4.88) *vs*. 2.46 1.92–3.24); *P* = 0.031; Table 2], suggesting that high *RAD54B* expression is associated with inferior prognostic outcome, specifically by causing postoperative distant metastasis. In contrast, RAD51 expression values were not significantly different between patients with and without distant recurrence [1.16 (0.81–1.79) *vs*. 1.07 (0.78–1.52); *P* = 0.69; Table 2]. According to these findings, we selected DRFS as the primary endpoint to further evaluate the prognostic ability of *RAD54B*.

### Clinicopathological features of *RAD54B* high and low groups in the training set

**Figure 1:**
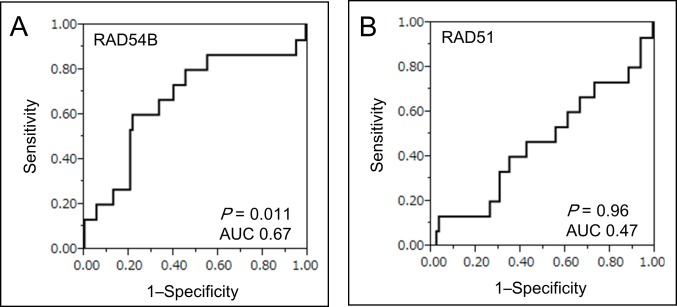
ROC curve analysis of *RAD54B* and *RAD51* expression **A.** ROC curve analysis using *RAD54B* expression values for distinguishing patients developing distant recurrence in the training set. The estimated optimal cut-off value of *RAD54B* expression was 3.63 (AUC 0.67, *P* = 0.011). **B.** ROC curve analysis using *RAD51* expression values for distinguishing patients developing distant recurrence in the training set (AUC 0.47, *P* = 0.96).

To evaluate the prognostic ability of *RAD54B* on DRFS, the optimal cut-off value of *RAD54B* expression for segregating DRFS was determined by receiver operating characteristic curve (ROC) analysis (Figure 1A). With the optimal cut-off value of 3.63, 108 stage I–III CRC patients in the training set were divided into two groups: low group (*n* = 79) and high group (*n* = 29). In contrast, we could not determine the optimal cut-off value of *RAD51* expression in the training set due to a low area under curve (AUC) score (Figure 1B).

Table 3 demonstrates a comparison of clinicopathological features of the *RAD54B* high and low groups in the training set (*n* = 108). Although the *RAD54B* high group had more advanced T stage (*P* = 0.027), factors including sex, age, tumor location, tumor size, cell differentiation, lymphatic invasion, venous invasion, preoperative tumor markers, UICC stage, and N stage were similar in both groups. Twenty-four patients (30.4%) in the *RAD54B* low group and nine patients (31.0%) in the high group received postoperative adjuvant chemotherapy consisting of 5-fluorouracil (5-FU)-based regimens (*P* = 0.95). During the median follow-up period of 50.7 (40.9–59.9) months, six patients (7.6%) in the *RAD54B* low group (*n* = 79) and nine patients (31.0%) in the high group (*n* = 29) developed distant recurrence (*P* = 0.0018).

**Table 3:** Comparison of clinicopathological factors between *RAD54B* high and low groups

Category		Training set (n = 108)		*P* value	Validation set (n = 71)		*P* value
		*RAD54B* Group			*RAD54B* Group		
		LOW (n = 79)	High (n = 29)		Low (n = 53)	High (n =18)	
		No (%)	No (%)		No (%)	No (%)	
Sex	Male	44 (55.7)	13 (44.8)	0.32	33 (62.3)	10 (55.6)	0.61
	Female	35 (44.3)	16 (55.2)		20 (37.7)	8(44.4)	
Age	<65	32 (40.5)	14 (48.3)	0.47	22 (41.5)	4 (22.2)	0.14
	≥65	47 (59.5)	15 (51.7)		31 (58.5)	14 (77.8)	
Tumor location	Colon	58 (73.4)	26 (89.7)	0.072	37 (69.8)	12 (66.7)	0.80
	Rectum	21 (26.6)	3(10.3)		16 (30.2)	6(33.3)	
Tumor size (mm)	<50	48 (60.8)	14 (48.3)	0.24	28 (52.8)	8 (4.4.4)	0.54
	≥50	31 (39.2)	15 (51.7)		25 (47.2)	10 (55.6)	
Cell differentiation	WD	41 (51.9)	12 (41.4)	0.33	26 (49.1)	6 (33.3)	0.25
	MD/PD	38 (48.1)	17 (58.6)		27 (50.9)	12 (66.7)	
Lymphatic invasion	Positive	24 (30.4)	12 (41.4)	0.28	19 (35.8)	3 (16.7)	0.13
	Negative	55 (69.6)	17 (58.6)		34 (64.2)	15 (83.3)	
Venous invasion	Positive	59 (74.7)	5 (17.2)	0.38	37 (69.8)	12 (66.7)	0.80
	Negative	20 (25.3)	24 (82.8)		16 (30.2)	6(33.3)	
Preoperative CEA	<5.0	41 (51.9)	16 (55.2)	0.76	29 (54.7)	7(38.9)	0.25
	≥5.0	38 (48.1)	13 (44.8)		24 (45.3)	II (61.1)	
Preoperative CA19-9	<37	69 (87.3)	24 (82.8)	0.88	43(81.1)	15 (83.3)	0.83
	≥37	10 (12.3)	5(17.2)		10 (18.9)	3(16.7)	
Tumor stage (UICC)	I	12 (15.2)	1(3.4)	0.17	7(13.2)	3(16.7)	0.88
	II	34 (43.0)	17 (58.6)		19 (35.8)	7 (38.9)	
	III	33 (41.8)	11 (37.9)		27 (50.9)	8(44.4)	
T stage	T1-2	21 (26.6)	2(6.9)	0.027	9(17.0)	4 (22.2)	0.62
	T3-4	58 (73.4)	27 (93.1)		44 (83.0)	14 (77.8)	
N stage	N0	46 (58.2)	18 (62.1)	0.72	26 (49.1)	11 (61.1)	0.38
	N1-2	33 (41.8)	11(37.9)		27 (50.9)	7(38.9)	
Adjuvant chemotherapy	Yes	24 30.4)	9 31.0)	0.95	23 (43.4)	6 (33.3)	0.45
	No	55 (69.6)	20 (69.0)		30 (56.6)	12 (66.7)	
Distant Recurrence	Yes	6 (7.6)	9 (31.0)	0.0018	7 (13.2)	7 (38.9)	0.024
	No	73 (92.4)	20 (69.0)		46 (86.8)	11 (61.1)	

WD = well differentiated; MD = moderately differentiated; PD = poorly differentiated.

### High *RAD54B* expression is an independent risk factor for distant recurrence

**Figure 2:**
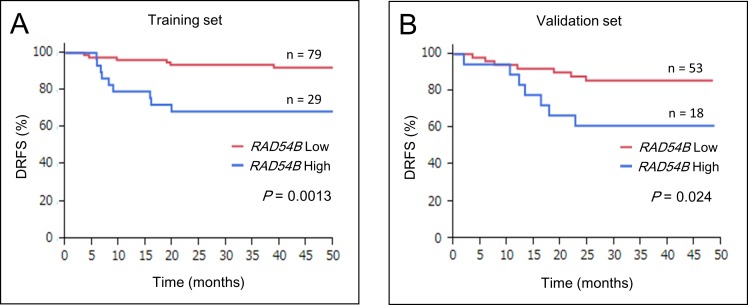
DRFS of *RAD54B* high and low groups **A.** Kaplan–Meier survival analyses of 108 stage I–III CRC patients in the training set revealed that the *RAD54B* high group had inferior DRFS compared with the low group (*P* = 0.0013). **B.** Kaplan–Meier survival analyses of 71 stage I–III CRC patients in the validation set revealed that the *RAD54B* high group had inferior DRFS compared with the low group (*P* = 0.024).

Kaplan–Meier survival analyses of 108 stage I–III CRC patients in the training set revealed that the *RAD54B* high group had inferior DRFS compared with the low group (estimated 3-year DRFS was 93.5% in the low group and 68.5% in the high group; *P* = 0.0013; Figure 2A). Multivariate analysis using the Cox proportional-hazards model showed that high *RAD54B* expression was the only independent prognostic factor associated with DRFS in the training set (hazard ratio, 4.31; 95% CI, 1.53–13.1; *P* = 0.0060; Table 4).

**Table 4:** Univariate and multivariate analysis of the factors associated with DRFS

Training set	Univariate analysis	HR	Multivariate analysis	*P* value
Factors	*P* value		95% CI	
*RAD54B* (High vs Low group)	0.0013	4.31	1.53 to 13.1	0.0060
T stage (T1-2 vs T3-4)	0.14	2.96	0.57 to 54.4	0.23
N stage (N0 vs N1-2)	0.14	2.44	0.88 to 7.23	0.088
Cell differentiation (WD vs MD/PD)	0.073	-	-	-
Lymphatic invasion (positive vs negative)	0.090	-	-	-
Venous invasion (positive vs negative)	0.33	-	-	-
Preoperative CEA (<5.0 vs ≥5.0)	0.51	-	-	-
Preoperative CA19-9 (<37 vs ≥37)	0.89	-	-	-
Age (<65 vs ≥65)	0.37	-	-	-
Location (Colon vs Rectum)	0.94	-	-	-
Sex (Male vs Female)	0.34	-	-	-
Tumor size (mm) (<50 vs ≥50)	0.49	-	-	-

Validation set	Univariate analysis	HR	Multivariate analysis	*P* value
Factors	*P* value		95% CI	
*RAD54B* (High vs Low group)	0.024	3.627	1.23 to 10.7	0.021
T stage (T1-2 vs T3-4)	0.18	3.432	0.66 to 63.1	0.17
N stage (N0 vs N1-2)	0.13	2.182	0.74 to 7.21	0.16
Cell differentiation (WD vs MD/PD)	0.34	-	-	-
Lymphatic invasion (positive vs negative)	0.54	-	-	-
Venous invasion (positive vs negative)	0.094	-	-	-
Preoperative CEA (<5.0 vs ≥5.0)	0.073	-	-	-
Preoperative CA19-9 (<37 vs ≥37)	0.19	-	-	-
Age (<65 vs ≥65)	0.41	-	-	-
Location (Colon vs Rectum)	0.74	-	-	-
Sex (Male vs Female)	0.71	-	-	-
Tumor size (mm) (<50 vs ≥50)	0.84	-	-	-

WD = well differentiated; MD = moderately differentiated; PD = poorly differentiated; HR = hazard ratio; CI = confidence interval.

### RAD54B expression in the validation set

Next, the *RAD54B* expression cut-off value of 3.63 determined in the training set was applied to the independent cohort of 89 CRC patients for validation. Seventy-one stage I–III CRC patients (excluding stage IV patients) in the validation set were stratified into the *RAD54B* low (*n* = 53) and high (*n* = 18) expression groups. Table 3 shows that clinicopathological factors including UICC stage, T stage, and N stage were not significantly different in the two groups. During the median follow-up period of 37.0 (28.5–46.0) months, seven patients (13.2%) in the *RAD54B* low group (*n* = 53) and seven patients (38.9%) in the high group (*n* = 18) developed distant recurrence (Table 3, *P* = 0.024). Kaplan–Meier survival analyses revealed that the *RAD54B* high group had inferior DRFS compared with the low group with (estimated 3-year DRFS was 85.7% in the low group and 61.1% in the high group; *P* = 0.024; Figure 2B). Multivariate analysis using the Cox proportional-hazards model showed that high *RAD54B* expression was an independent prognostic factor associated with DRFS even in the validation set (hazard ratio, 3.63; 95% CI, 1.23–10.7; *P* = 0.021; Table 4).

### *RAD54B* expression is inversely correlated with *CDKN1A* expression

**Figure 3:**
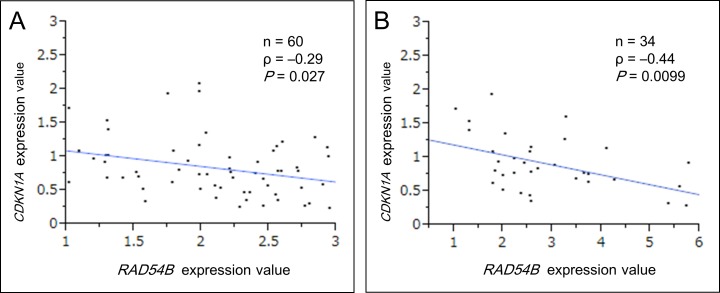
Scatter plot analysis between *RAD54B* and *CDKN1A* expression **A.** A weakly significant negative correlation was found between *RAD54B* and *CDKN1A* expression values in wild-type p53 samples with *RAD54B* expression values ranging between 1.00–2.95 (ρ = −0.29, *P* = 0.027). **B.** A moderately significant negative correlation was found between *RAD54B* and *CDKN1A* expression values in wild-type p53 samples with Arg72 variant, provided RAD54B expression values were under 6.00 (ρ = −0.44, *P* = 0.0099).

We next studied the relationship between *RAD54B* and p53 functions in clinical CRC samples. Because p53 activates *CDKN1A* gene transcription and its expression reflects the functional status of p53 protein [25, 26], the *CDKN1A* expression was measured and compared with that of *RAD54B*. This analysis was limited to samples without any p53 hotspot mutations observed in CRC because most of such p53 mutants lack the normal transcriptional activities [27, 28]. Using sequencing analysis of the p53 gene, 92 samples without p53 hotspot mutations were extracted from the combined training and validation set (*n* = 212). Our data showed that *CDKN1A* expression tends to decrease remarkably in CRC samples which expression of *RAD54B* are within an approximately 3-fold increase compared with normal mucosa. Particularly, in samples with RAD54B expression values ranging between 1.00–2.95, Spearman's rank-order correlation demonstrated a significant weakly negative correlation between *CDKN1A* and *RAD54B* expression (*ρ* = −0.29, *P* = 0.027; Figure 3A).

The degradation of p53 caused by RAD54B requires the interaction between p53 and MDM2 [13]. A study reported that p53 has a sequence polymorphism resulting in either Pro or Arg at amino acid position 72, and that although both variants can equivalently transactivate *CDKN1A*, the Arg72 variant undergoes ubiquitination markedly better than the Pro72 variant via greater interaction with MDM2 [29]. Therefore we analyzed the sequence of amino acid position 72 and the correlation between *RAD54B* and *CDKN1A* expression. The analysis limited to 34 samples with Arg72 in 92 samples without p53 hotspot mutations revealed that there was a more remarkably significant negative correlation between *RAD54B* and *CDKN1A* expression provided *RAD54B* expression values were under 6.00 (*ρ* = −0.44, *P* = 0.0099; Figure 3B).

## DISCUSSION

The present study is the first report demonstrating the utility of *RAD54B* as a prognostic biomarker in CRC patients. Human *RAD54B* was identified in 1999 as a homolog of RAD54. Biochemical studies have shown that RAD54B plays an important role in the DNA repairing system by HR [6-9]. However, few studies have focused on the clinical importance of RAD54B thus far. In this study, we confirmed that *RAD54B* expression was elevated in the majority of the CRC tissues compared with corresponding normal mucosa (116/123 in the training set, 94.3%). Based on this finding, we examined the impact of RAD54B expression on the clinical outcome of CRC patients. Our results revealed that CRC patients with high *RAD54B* expression had significantly inferior DRFS compared with those with low *RAD54B* expression. Multivariate analyses using Cox proportional-hazards regression model demonstrated that high *RAD54B* expression was an independent risk factor for distant recurrence in stage I–III CRC patients in both the training and validation sets. These results clearly demonstrate the prognostic value of *RAD54B* expression in CRC patients.

We also analyzed *RAD51* expression in the same training set. Although RAD51 is another central component in the HR pathway [15-18], the association of *RAD51* expression and cancer prognosis remains controversial. Several studies reported that RAD51 overexpression was associated with poor prognosis in various cancers such as non-small-cell lung carcinoma, head cancers, esophageal squamous cell carcinoma, breast cancer, melanoma, and CRC [19-24]. In contrast, two studies targeting human glioblastoma and breast cancer reported the opposite results that high *RAD51* expression was associated with better prognostic outcome [30, 31]. In our study, *RAD51* expression was elevated in 60.2% (74/123 in the training set) of CRC samples compared with normal mucosa. This percentage was similar to previous studies [19-24]. However, *RAD51* expression was not predictive of disease prognosis such as DRFS in our cohort. Our result implies that the prognostic ability of *RAD54B* might be better than that of *RAD51* in CRC patients. On the other hand, the discrepancy of our results compared with previous reports might arise from the difference in the analysis method, e.g., RAD51 expression was examined using immunohistochemistry in most previous studies, whereas we assessed *RAD51* expression using a real-time PCR.

Meanwhile, the difference of clinical influence between *RAD54B* and *RAD51* expression implies another pathway other than the HR, where RAD54B functions as a negative prognostic factor. Based on our *in vitro* study that RAD54B enhances p53 protein degradation via the MDM2–MDMX ubiquitin–ligase complex [13], we analyzed *CDKN1A* expression, a target gene of p53, to elucidate the biological role of RAD54B in clinical CRC tissues. The analysis limited to CRC samples with Arg72 showed a significant inverse correlation between *RAD54B* and *CDKN1A* expression. This result partially supports the mechanism that RAD54B functions via the degradation of p53 protein function in clinical CRC tissues. However, we could not detect a significant correlation between *RAD54B* and *CDKN1A* expression in samples with remarkably high RAD54B expression, implying the presence of another unknown mechanism causing poor prognosis.

Several studies reported that RAD51 overexpression induced tumor resistance to ionizing radiation and anticancer drugs *in vitro* [32-34]. As for RAD54B, we previously found that xenografts derived from RAD54B knock out HCT116 cells grew more slowly than that from wild-type HCT116 cells after either oxaliplatin or 5-FU treatment in nude mice [13]. These results suggests the possibility that the effect of chemotherapy attenuates by RAD54B overexpression, and suggests the potential therapeutic target such as blocking the RAD54B overexpression to reinforce the effect of the chemotherapy. Unfortunately, in the present study, we were not able to prove statistically the association between *RAD54B* expression and the effect of adjuvant chemotherapy consisting mainly of 5-FU based regimens probably because of our limited number of patients. We will further examine the effect of *RAD54B* expression on chemotherapy by accumulating more CRC patients.

Limitations to the present study should be noted. First, the samples were obtained from a single institution and the sample size was not large enough for subgroup analysis. Second, the follow-up period may have been relatively short. Third, this was a retrospective study. A large, prospective cohort study would be desirable to determine the clinical usefulness of RAD54B more accurately.

In conclusion, this study is the first report clinically demonstrating the importance of *RAD54B* as a prognostic biomarker in CRC patients. An increased expression level of *RAD54B* may serve as an independent predictor of poor outcome in CRC patients treated with surgical resection, particularly for distant metastasis. Postoperative recurrence risk stratification by *RAD54B* expression will be beneficial for more individual cancer strategies.

## MATERIALS AND METHODS

### Collection of tissue samples and clinical data

A total of 212 consecutive CRC samples were analyzed. All patients undergoing surgical resection were pathologically diagnosed with CRC at University of Tokyo Hospital, Tokyo, Japan. Samples obtained between 2008 and 2010 were included in the training set (*n* = 123), whereas those obtained between 2011 and 2012 were included in the validation set (*n* = 89). To prevent the influence of preoperative therapy on the targeted gene expression in resected specimens, patients receiving any preoperative chemotherapy or radiotherapy were excluded from the study.

A detailed database containing clinical and pathological information was provided for statistical analysis, and survival data were acquired from clinical charts. The cancer histological grade and clinical stage were identified in accordance with the 7th edition of the TNM classification of Union for International Cancer Control (UICC). The study protocol was approved by the ethics committee of The University of Tokyo Hospital, and written informed consent was obtained from all partici­pating patients.

### Total RNA extraction and cDNA synthesis

Tumor tissue and corresponding normal mucosa were first immersed in RNAlater Tissue Protect Tubes (QIAGEN) overnight at 4°C and stored at −20°C until analysis.

Total RNA was extracted from tissue samples using TRIzol Reagent (Life Technologies) and subsequently treated with DNase I (TAKARA BIO INC). Complementary DNA (cDNA) was synthesized from total RNA using PrimeScript RT Master Mix (TAKARA BIO INC) and used as a template for real-time PCR and cDNA sequencing. Each procedure was performed according to the manufacturer's instructions.

### Real-time PCR

Gene expression of *RAD54B*, *RAD51*, *CDKN1A*, and TATA-binding protein (*TBP*) were analyzed by real-time PCR using a 7500 Fast Real-Time PCR system (Life Technologies), with KAPA SYBR FAST qPCR Master Mix (KAPA Biosystems), according to the manufacturer's instructions. The following sets of primers were used: *RAD54B*, 5′-TCCAGGTCTGAATGAAGAGATTAC-3′ and 5′-TCTAGTACTTTCTTCACTAGGCAG-3′ *RAD51*, 5′-TATCCAGGACATCACTGCCA-3′ and 5′-GGTGAAGGAAAGGCCATGTA-3′ *CDKN1A*, 5′-GACTCTCAGGGTCGAAAACGG-3′ and 5′-GCGGATTAGGGCTTCCTCTTG-3′ and *TBP*, 5′-TGCTGCGGTAATCATGAGGATA-3′ and 5′-TGAAGTCCAAGAACTTAGCTGGAA-3′.

All measurements were performed in triplicates and mean cycle threshold (Ct) values of both tumor tissue and corresponding normal mucosa were calculated for the expression analysis. Gene expression analysis was performed using the ΔΔCt method (*RAD54B* and *RAD51*) or the standard curve method (*CDKN1A*) [35]. *TBP* was used as an internal control to normalize gene expression values for each gene expression analysis.

The ΔCt value represents the difference between the Ct value of the target gene (*RAD54B* and *RAD51*) and that of *TBP* in the same sample, whereas the ΔΔCt value indicates the difference between the average ΔCt value of the tumor tissue and that of the corresponding normal mucosa. The targeted gene expression value was obtained as 2^−ΔΔCt^ and used in the following analyses.

### cDNA sequencing of the p53 gene

For sequencing of p53 gene transcripts, cDNA of the tumor tissue was amplified with PrimeSTAR HS DNA Polymerase (TAKARA BIO INC). Primer sets covering the regions from the first ATG to Gln167 and from Arg156 to Pro301 were used to detect the amino acid polymorphism at position 72 and CRC hot-spot mutations, respectively [29, 36]. Direct cDNA sequencing of each PCR product was performed using BigDye terminator v3.1 (Life Technologies) on a 3100 Genetic Analyzer (Life Technologies). The following sets of primers were used: ATG–Gln167, 5′-GTGACACGCTTCCCTGGAT-3′ and 5′-CTCACAACCTCCGTCATGTG-3′ and Arg156–Pro301, 5′-GTGCAGCTGTGGGTTGATT-3′ and 5′-CAGTGCTCGCTTAGTGCTCC-3′.

### Statistical analysis

All statistical analyses were conducted using JMP pro version 10 software packages (SAS Institute). Continuous variables were presented as medians (inter-quartile ranges), and they were analyzed using Kruskal–Wallis test (multiple groups) or Mann–Whitney U test (two groups). Categorical variables were presented as numbers (%) and analyzed using Pearson's chi-squared test or Fisher's exact test, as appropriate. For measuring the strength of association between expression values of targeted genes, Spearman's rank-order correlation was used. Kaplan–Meier survival analysis was adopted for DRFS rate analysis, and survival differences between patients groups were evaluated using the log-rank test. DRFS was defined as the time from surgery to distant metastasis. Multivariable analyses for DRFS were performed using the Cox proportional-hazards regression model, and results were presented as hazard ratios with 95% confidence intervals. Probability values (*P*) < 0.05 were considered statistically significant.
